# The DNA Story, Part III

**DOI:** 10.1371/journal.pbio.0020067

**Published:** 2004-03-16

**Authors:** Paul Doty

## Abstract

Maurice Wilkins's autobiography provides an engaging perspective of the events leading to the discovery of the structure of DNA

[Fig pbio-0020067-g001]As the year-long celebration of the 50^th^ anniversary of the discovery of the structure of DNA came to an end, the engaging autobiography of one of the participants further enlivened the drama of this event. Maurice Wilkins, now 87, postpones the account of his involvement in the DNA affair until the second half of the book. Recounting his background and interesting life before DNA (34 years) in plain but telling sentences brings to life a character that is almost as much out of the ordinary as those of the more flamboyant James Watson and Francis Crick.

**Figure pbio-0020067-g001:**
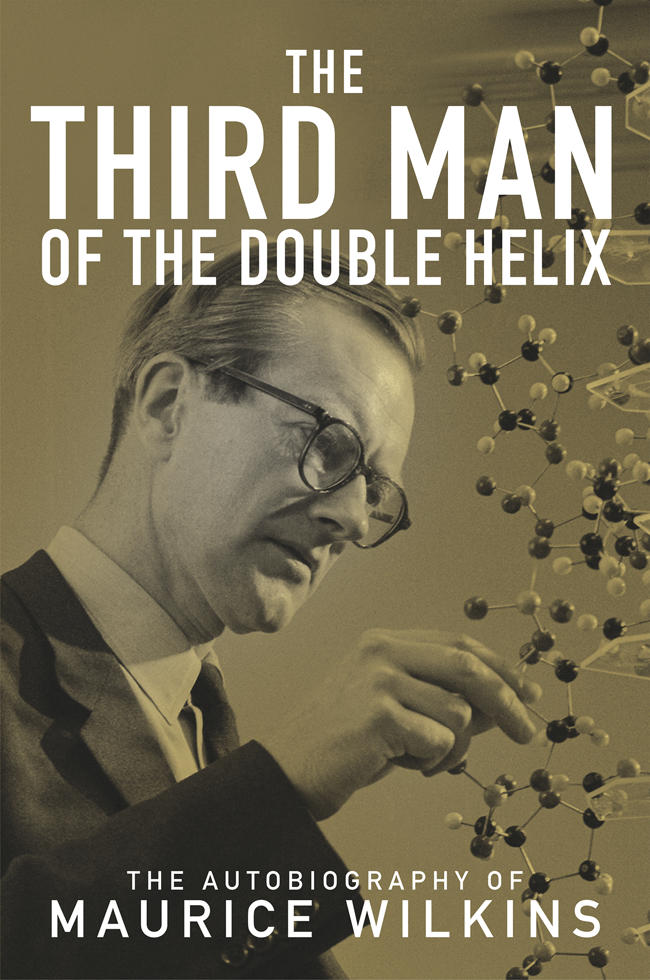


Wilkins' first six years in New Zealand (a Garden of Eden) were followed by a long, vividly described trip to England, where the family eventually settled in Birmingham. His boyhood was marked by immersion in astronomy and telescope-making, but saddened by the painful illness of his sister. Success in school physics was the key for getting into Cambridge, where he reveled in the world of Ernest Rutherford, Mark Oliphant, and John Bernal. Given his leftist leanings, it was inevitable that Wilkins would become involved in the pacifist movement in Cambridge, with its close connection to the Communist Party. Perhaps too much involvement led to a low degree grade in 1938 and no hope of remaining at Cambridge. Instead, he returned to Birmingham and joined the Luminescence Lab being established by John Randall, a man with whom he would be closely connected for many decades. The work there contributed to Randall's scheme for making radar practical in air defense—the cavity magnetron that may have turned the course of World War II.

Early in 1944, Oliphant, then at Birmingham, left to work on the atomic bomb at Berkeley and took Wilkins along. Life in Berkeley was exciting, but beneath the excitement of bomb work and mixed feelings upon its success at Hiroshima, Wilkins read Erwin Schrodinger's *What Is Life?* Along with others who were to unravel the secrets of DNA, this planted the seed. When, after three transitional years, Randall became head of Kings College London's physics department and director of a biophysics research unit sponsored by the Medical Research Council (MRC), Wilkins was his deputy. The attack on DNA structure soon began.

That X-ray diffraction might play a major role in this search rested on two pillars unique to England. One was the British lead in using X-ray diffraction to determine molecular structures—a crown jewel built on the work of the Braggs (father and son), Bernal, and Dorothy Hodgkin. The other was the pre-World War II work of William Astbury in showing that DNA fibers displayed some crystallinity that, if developed, might be the basis of helping to determine the structure. Wilkins confides that in 1950 he knew little of how such X-ray analysis might be done. But in that year he was presented with an opportunity in the form of samples of carefully prepared calf DNA, given to him by a Swiss chemist, Rudolf Signer. With this DNA, much better fibers could be obtained and much sharper diffraction diagrams emerged.

The exploitation of this advance, however, became mired in a colossal error in Randall's management of the group. Without telling Wilkins, he wrote to Rosalind Franklin, who was on her way to join the DNA effort, that Wilkins was withdrawing from DNA work and that she would take over. Unaware of this, Wilkins and Alec Stokes continued their work and reported at a meeting in Cambridge in July 1951 that DNA chains were probably in a helical conformation with a diameter of 20 Å. At the close of the meeting, Franklin assailed Wilkins, saying that he should stop his DNA work (as Randall had written would be the case). Understandably, but regrettably, the two groups continued working in isolation from each other.

Matters worsened. In October, Watson arrived at Cambridge and set up DNA structure studies with Crick. They quickly arrived at a three-stranded helical structure. But Franklin and Wilkins soon demolished it. Likewise, a three-stranded model at Kings College had a very short life. As if to trump these failures, Bragg at Cambridge and Randall at Kings agreed that DNA studies at Cambridge should stop and that the work should continue only at Kings. Mismanagement and noncooperation were taking their toll. Franklin was moving toward a two-stranded structure, but away from helices. Indeed, in mid-1952 she initiated a discussion with an announcement about the death of the helix. Mysteriously, she put aside a striking photo of the diffraction pattern of B-DNA (one of the two major structural forms of DNA) that emerged in early 1953 as a perfect signature of the helical form. But 1952 continued downhill. Even Wilkins stopped DNA work that November.

Suddenly, in the new year, life returned to the DNA effort. Linus Pauling had just published a structure (three-stranded) that did not long survive, but the entrance of the world's leading structural chemist into the race reawakened everyone to the centrality of DNA structure. In January, Raymond Gosling gave to Wilkins the very well-oriented diffraction photo of B-DNA that he and Franklin had taken in July 1952. Wilkins assumed that it was given to him to do as he wished; a few days later, he showed it to Watson. Though hardly an expert in X-ray diffraction, Watson sensed that it was strong evidence for helices and sketched it for Crick on his return to Cambridge. Later that January, Franklin announced she would be moving from Kings College to Birkbeck College to join Bernal's group. In giving her final seminar, she switched from her earlier insistence that B-DNA was nonhelical, but did not show the photo that gave the strongest evidence for helicity. This shift put Franklin in a position to move forward on the structure of DNA, but without others' resorting to model building, the goal would have remained elusive.

Finally, in mid-February, Max Perutz, who was a member of the MRC committee overseeing the Biophysics Unit at Kings College, passed on to Crick his copy of a report from that unit. This report contained Franklin's results that the phosphates were on the outside and that the A-form of DNA had a special crystalline arrangement called the monoclinic C2 space group. From his work with proteins, Crick saw immediately that the chains in the helical structure must be antiparallel and that there were probably two chains entwined. Watson used other data in the report to deduce that there were indeed two chains, not three or four. Erwin Chargaff had recently shown that in the base composition of all DNAs examined, adenines and thymines as well as guanines and cytosines are equal, i.e., A = T and G = C. Now released from the ban on DNA studies, Watson and Crick engaged in a frantic search using model building. They found a unique way to fit the bases in the structure by pairing, and by March 7 they had the double-helix model constructed: it obeyed the Chargaff ratios, it fit the X-ray data for B-DNA, and it provided a rational way to encode and transmit genetic information to subsequent generations.

Wilkins was invited to view the model in Cambridge. He found it stunning. Watson asked him to be a coauthor of the paper. Wilkins, true to his character, declined, as he had not been involved in the final monumental stage. Back in London, Franklin had already moved to Birkbeck. She received the news of the discovery with equanimity. But a later examination of her notebooks showed that she had moved to favor helices and a two-chain (or possibly a one-chain) model.

With the rather complicated story of the greatest discovery in biology in the century now reasonably complete, what is one to make of it? There are many answers. I will mention only three.

The first is the key role played by model building. In fiber diffraction there is not enough information, by orders of magnitude, to locate every atom, as would be possible in diffraction by perfect crystals that give thousands of sharp reflections. Instead, the fiber diagram can only provide cues and some specifics, such as the repeat distance. Model building is a way of bringing into the picture previously determined bond distances and bond angles of components such as the purine and pyrimidine bases and the sugars that are unavailable from the fiber diagram. That this was not seen at Kings College left the researchers there well behind in a field that they had pioneered.

A second lesson is the importance of bringing the full knowledge of single crystal analysis to fiber diagram interpretation. That Franklin and Wilkins missed noting that the monoclinic C2 space group meant that the chains in the fiber had to be antiparallel robbed them of an important clue to the structure.

And third, the management of the Biophysics Unit at Kings College was a recipe for failure. Riddled by secrecy, diffuse lines of authority, the absence of strategies, and a lack of open congeniality, all so well described by Wilkins, who refers to it as Randall's Circus, this unit is a model of how not to succeed in group research.

DNA research continued at Kings College in a gradually improving environment: important details were worked out. But there was no real renewal, such as aiming at how DNA is configured to accommodate proteins in the nucleus. Wilkins enjoyed being included in the subsequent awards—the Lasker and the Nobel prizes. With Crick, he was annoyed by Watson's rendering of events in *The Double Helix*. The final chapter of his own autobiography addresses the criticism that some have leveled against his cold relation with Franklin, but also his happiness in newfound family life. Research gradually gave way to the pursuit of pacifist goals in a number of organizations and to the popularization of science. His has been a useful life, a part of which contributed to the great revolution in biology. It is good to have the insight that this book presents in a candid and personal way.

